# Association of dual-energy CT-derived quantitative parameters with hydroxyproline levels and pulmonary function in nonspecific interstitial pneumonia

**DOI:** 10.3389/fimmu.2026.1862309

**Published:** 2026-09-07

**Authors:** Zhenghua Zhang, Jiashu Li, Feng Yuan, Deyan Li, Qian Xiong, Wenyan Wei, Xiao Cui, Jinzhi Chenhuang, Daying Fen, Jianqing Zhang, Dan Han

**Affiliations:** The First Affiliated Hospital of Kunming Medical University, Kunming, China

**Keywords:** dual-energy CT, hydroxyproline, nonspecific interstitial pneumonia, pulmonary fibrosis, pulmonary function

## Abstract

**Background:**

This study evaluated the feasibility of dual-energy CT (DECT) for assessing the correlation between hydroxyproline (HYP) levels in lung tissue of patients with nonspecific interstitial pneumonia (NSIP) and lung volume and pulmonary function.

**Methods:**

We retrospectively analyzed 99 NSIP patients (51 cellular, 48 fibrotic) and 40 controls. All underwent DECT imaging. Lung HYP‑equivalent density (HYP‑ED) and volume were quantified using spectral post-processing. Pulmonary function parameters, including FVC% Pred and DLCO% Pred, were collected. Intergroup differences, correlations, and diagnostic performance were assessed.

**Results:**

Lung volume was higher in controls (3797.40 ± 1155.68 cc) than in cellular (2796.88 ± 334.03 cc) and fibrotic NSIP (2156.76 ± 423.40 cc; *P* < 0.001). HYP‑ED was lower in controls (206.29 ± 22.40 mg/cm^3^) than in cellular (264.31 ± 24.99 mg/cm^3^) and fibrotic NSIP (327.12 ± 36.26 mg/cm^3^; *P* < 0.001), and lower in cellular than fibrotic NSIP (*P* < 0.001). FVC% Pred and DLCO% Pred were higher in cellular NSIP (75.98 ± 4.07% and 69.96 ± 4.41%) than in fibrotic NSIP (71.44 ± 5.49% and 64.58 ± 3.38%; *P* < 0.05). HYP‑ED content showed significant negative correlations with lung volume and pulmonary function (all *P* < 0.01). HYP‑ED demonstrated excellent diagnostic performance for distinguishing NSIP subtypes (AUC 0.977), with 91.8% sensitivity and 92.9% specificity.

**Conclusions:**

DECT enables noninvasive quantification of lung HYP-ED in NSIP. HYP‑ED correlates with disease severity and may serve as a promising imaging biomarker for fibrosis assessment and monitoring.

## 1 Introduction

Nonspecific interstitial pneumonia (NSIP) represents a distinct histopathological subtype of interstitial lung disease (ILD), characterized by a temporally and spatially homogeneous pattern of interstitial inflammation and fibrosis within the pulmonary parenchyma. Current classification systems stratify NSIP into cellular and fibrotic subtypes based on histopathological characteristics and the degree of fibrotic involvement, which exhibit marked differences in clinical outcomes, therapeutic responsiveness, and disease trajectories (1–3). The pathophysiological mechanisms underlying NSIP involve sustained alveolar epithelial damage, chronic inflammatory cascades, and subsequent activation of fibroblasts and myofibroblasts, culminating in excessive deposition of collagen and extracellular matrix components. This aberrant remodeling process ultimately leads to progressive deterioration of pulmonary function (4). Hydroxyproline (HYP), a collagen-specific amino acid, serves as a robust biochemical surrogate for collagen deposition and is widely utilized as a quantitative marker of tissue fibrosis (5, 6).

Although surgical lung biopsy remains the gold standard for histopathological subtyping and fibrosis severity assessment in NSIP, its invasive nature carries inherent risks, including hemorrhage and pneumothorax. Furthermore, even in NSIP with relatively uniform lesion morphology, the limited volume of biopsy specimens cannot fully represent the overall fibrotic burden of the entire lung, resulting in sampling bias. Repeated biopsies are also clinically impractical for longitudinal disease monitoring (7). Conventional chest CT and pulmonary function test (PFT) remain the mainstay of clinical evaluation, but morphological assessment is largely subjective and semi-quantitative, and functional changes often lag behind structural tissue remodeling (8, 9). To date, there remains a lack of noninvasive, reproducible, and quantitative methods that directly reflect *in vivo* collagen deposition levels in lung tissue, creating an unmet need for precise fibrosis stratification, therapeutic response assessment, and prognostic evaluation in NSIP.

Dual-energy CT (DECT) acquires attenuation data at distinct energy levels, enabling material decomposition and quantitative analysis based on energy-dependent variations in X-ray attenuation. This technique facilitates non-invasive tissue characterization and has demonstrated promising utility in pulmonary perfusion imaging, material differentiation, and quantitative tissue analysis (10). As such, DECT represents a novel imaging modality for the quantitative assessment of interstitial lung abnormalities (11). Prior work has validated the reliability of DECT for quantifying specific tissue components in the lung, such as iron oxide deposition in pneumoconiosis, using dedicated base material pairs (12). However, DECT-based quantification of pulmonary HYP‑equivalent density (HYP‑ED) has not been systematically applied or validated in NSIP populations, and its associations with structural lung volume changes and functional impairment remain unclear.

In this study, we sought to quantify pulmonary HYP-ED using DECT in patients with NSIP and healthy controls, examine its correlation with lung volume and pulmonary function parameters, and evaluate its diagnostic accuracy in distinguishing cellular from fibrotic NSIP. Our findings aim to establish an imaging-derived biomarker for the non-invasive evaluation of pulmonary fibrosis in NSIP.

## 2 Materials and methods

### 2.1 Study population

This retrospective study enrolled 152 patients diagnosed with NSIP through multidisciplinary discussion (MDD) at our institution between January 2024 and January 2026. The inclusion criteria comprised: (1) completion of DECT chest scanning, (2) availability of PFT results, and (3) an interval of ≤ 4 weeks between PFT and DECT. Exclusion criteria included: (1) concurrent pulmonary conditions that could confound quantitative lung parenchymal analysis (emphysema, active pulmonary infections, primary lung malignancy, and prior lung resection surgery, etc.), (2) suboptimal CT image quality precluding quantitative assessment, and (3) incomplete clinical records.

**Figure 1:**
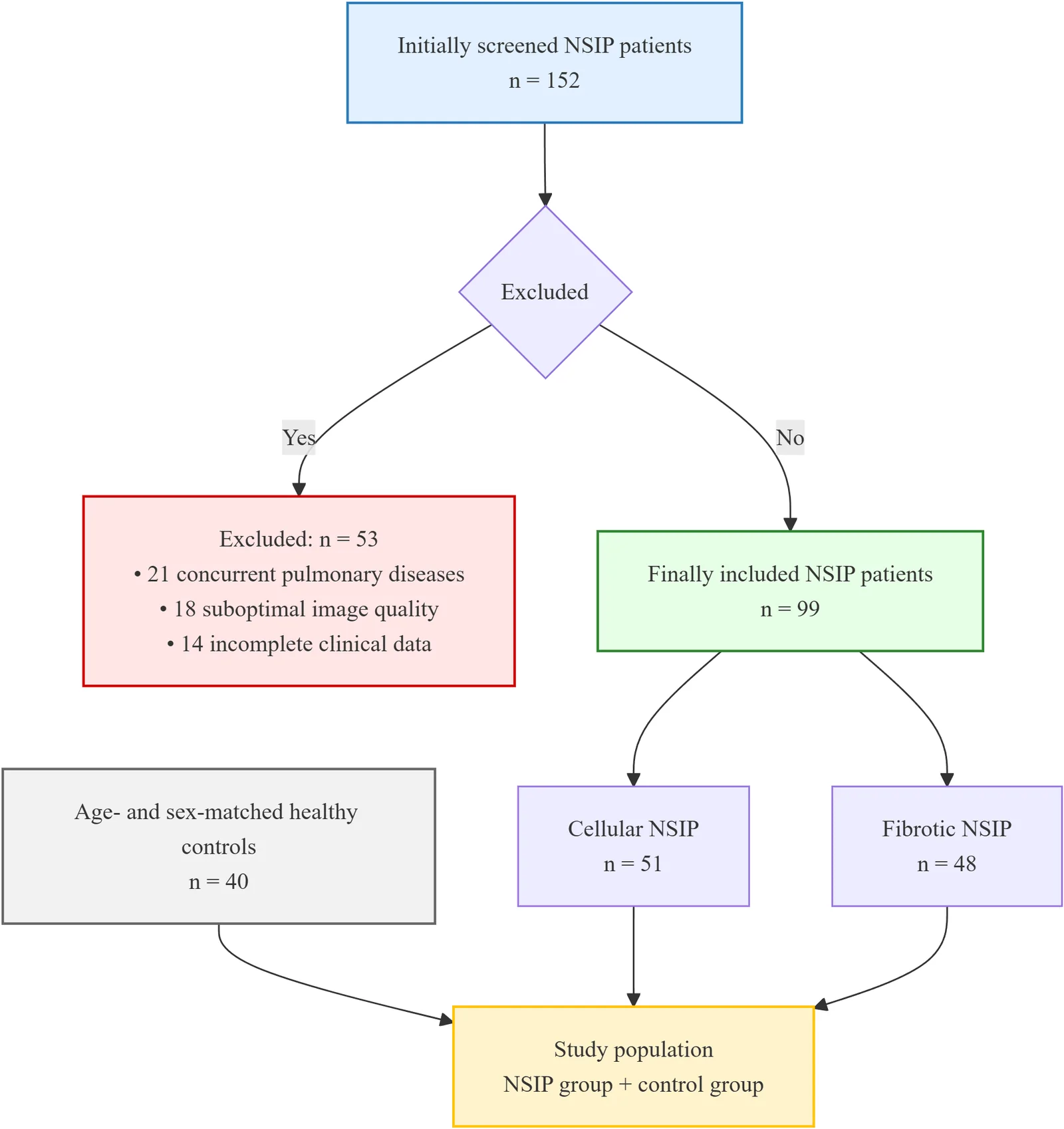
Patient recruitment flowchart.

After screening, 99 NSIP patients were included in the final analysis, comprising 51 cellular subtype and 48 fibrotic subtype cases. Additionally, 40 age- and sex-matched healthy controls from the same period were recruited. A patient recruitment flowchart is provided in **Figure 1**.

All NSIP diagnoses were formally established via institutional MDD consisting of respiratory physicians, rheumatology and immunology physicians, radiologists, and pathologists, in accordance with the 2013 ATS/ERS IIP diagnostic criteria. Subtype differentiation was based on integrated histopathological and clinical-radiological evidence:

1. Cellular NSIP: histologically dominated by interstitial inflammatory cell infiltration with minimal fibrosis; radiologically dominated by diffuse ground-glass opacity with absent or minimal fine reticulation and no significant traction bronchiectasis.
2. Fibrotic NSIP: histologically characterized by marked interstitial fibrosis with variable inflammatory infiltration; radiologically demonstrating prominent reticular opacity, traction bronchiectasis, and architectural distortion, with or without ground-glass opacity.

Imaging pattern evaluation followed Fleischner Society ILD classification standards. Two board-certified chest radiologists with >10 years of ILD experience independently reviewed all CT images, blinded to MDD subtype assignments; disagreements were resolved by consensus.

Among the 99 patients, 28 underwent lung biopsy (21 transbronchial cryobiopsy, 7 surgical lung biopsy) with histopathologically confirmed NSIP subtype; the remaining 71 received a clinicoradiological MDD diagnosis per guideline recommendations. Etiologically, 62 cases were idiopathic NSIP, and 37 were connective tissue disease-associated NSIP (15 Sjogren’s syndrome, 12 rheumatoid arthritis, 10 systemic sclerosis).

Among the 99 patients with NSIP, 41 had not received any immunosuppressive or anti-fibrotic therapy before the CT examination, while 58 had received pharmacological therapy prior to DECT acquisition. The regimens were as follows: 32 patients received glucocorticoid monotherapy, 18 received a combination of glucocorticoids and immunosuppressants, and 8 were treated with anti-fibrotic drugs (pirfenidone or nintedanib).

Written informed consent was obtained from all participants, and the study protocol was approved by the Ethics Committee of the First Affiliated Hospital of Kunming Medical University (NO. 2026L-85).

### 2.2 CT imaging and post-processing

Thoracic DECT was performed using a GE Revolution scanner with the following parameters: non-contrast Gemstone Spectral Imaging (GSI) mode, tube voltage 120 kV, automatic tube current modulation (200–450 mA), 0.625 mm slice thickness, pitch 0.992, and 512×512 matrix. Scans were acquired from the lung apex to base during end-inspiration in the supine position.

Post-processing was conducted on an ADW 4.7 GSI Viewer workstation. HYP-ED (mg/cm^3^) was derived from dual-energy material decomposition using hydroxyproline and calcium as basis materials. It represents the equivalent density of HYP-like attenuating components in each voxel of lung parenchyma, rather than direct biochemical concentration. The total lung volume (cc) was also obtained.

The extent of disease involvement on pulmonary CT was assessed using a visual semi-quantitative five-lobe method. Two physicians blinded to clinical data independently reviewed the thin-section CT lung window images. Both lungs were anatomically divided into five lobes, and the percentage of disease involvement relative to each lobe’s volume was estimated lobe by lobe. Scores were assigned using a five-point scale: 0 (no involvement), 1 (< 25%), 2 (25%–49%), 3 (50%–75%), and 4 (> 75%). The total disease score was obtained by summing the scores of all five lobes, resulting in a range of 0 to 20.

### 2.3 Pulmonary function testing

Key parameters analyzed included: (1) forced vital capacity percent predicted (FVC% Pred) and (2) diffusing capacity for carbon monoxide percent predicted (DLCO% Pred).

### 2.4 Statistical analysis

Data were analyzed using SPSS 22.0 (IBM Corp.). The normality of continuous variables was assessed using the Shapiro-Wilk test. Continuous variables with normal distribution were expressed as mean ± standard deviation. Intergroup comparisons were performed using one-way ANOVA with *post-hoc* Tukey tests for multiple comparisons. Categorical variables were presented as frequencies (percentages) and compared using the chi-square (χ²) test or Fisher’s exact test when expected cell counts were < 5. Interobserver agreement was evaluated using the intraclass correlation coefficient (ICC). Pearson correlation analysis evaluated associations between HYP‑ED, lung volume, and PFT parameters. Univariate linear regression was additionally performed to quantify the magnitude of change per unit HYP-ED increase. Receiver operating characteristic (ROC) curve analysis assessed the discriminative performance of these parameters in distinguishing NSIP subtypes. A two-tailed p-value <0.05 was considered statistically significant.

## 3 Results

### 3.1 Baseline characteristics comparison

Detailed baseline characteristics, including age, gender, body mass index (BMI), disease etiology, comorbidities, lesion extent score, biopsy status, and prior treatment, are summarized in **Table 1**.

**Table 1:** Detailed baseline characteristics of the study population.

Parameter	Control (n=40)	Cellular NSIP (n=51)	Fibrotic NSIP (n=48)	*P*
Age (years)	52.4±8.2	53.5±7.6	55.6±8.4	0.165
Male, n (%)	20(50.0)	21(41.2)	21 (43.8)	0.695
Female, n (%)	20(50.0)	30(58.8)	27(56.2)	0.695
BMI (kg/m²)	20.3 ± 2.0	19.2 ± 2.1	18.9 ± 1.9	0.058
Smoking, n (%)	11(27.5)	12(23.5)	14(29.2)	0.809
Hypertension, n (%)	6(15.0)	10(19.6)	9(18.8)	0.839
Diabetes, n (%)	4(10.0)	5(9.8)	5(10.4)	0.995
Idiopathic NSIP, n (%)	/	33 (64.7)	29 (60.4)	0.659
CTD - NSIP, n (%)	/	18(35.3)	19(39.6)	0.659
Lesion extent score (0–20)	/	10.3±2.2	11.8±2.7	0.075
Lung biopsy performed, n (%)	/	16(31.4)	12(25.0)	0.482
Prior therapy, n (%)	/	28(54.9)	30(62.5)	0.443
Honeycombing present, n (%)	/	0(0)	6(12.5)	0.027

Interobserver agreement for the semi-quantitative score was evaluated by ICC, with an ICC value of 0.896 indicating excellent reliability.

### 3.2 Comparison of lung volume and HYP-ED among groups

**Figure 2:**
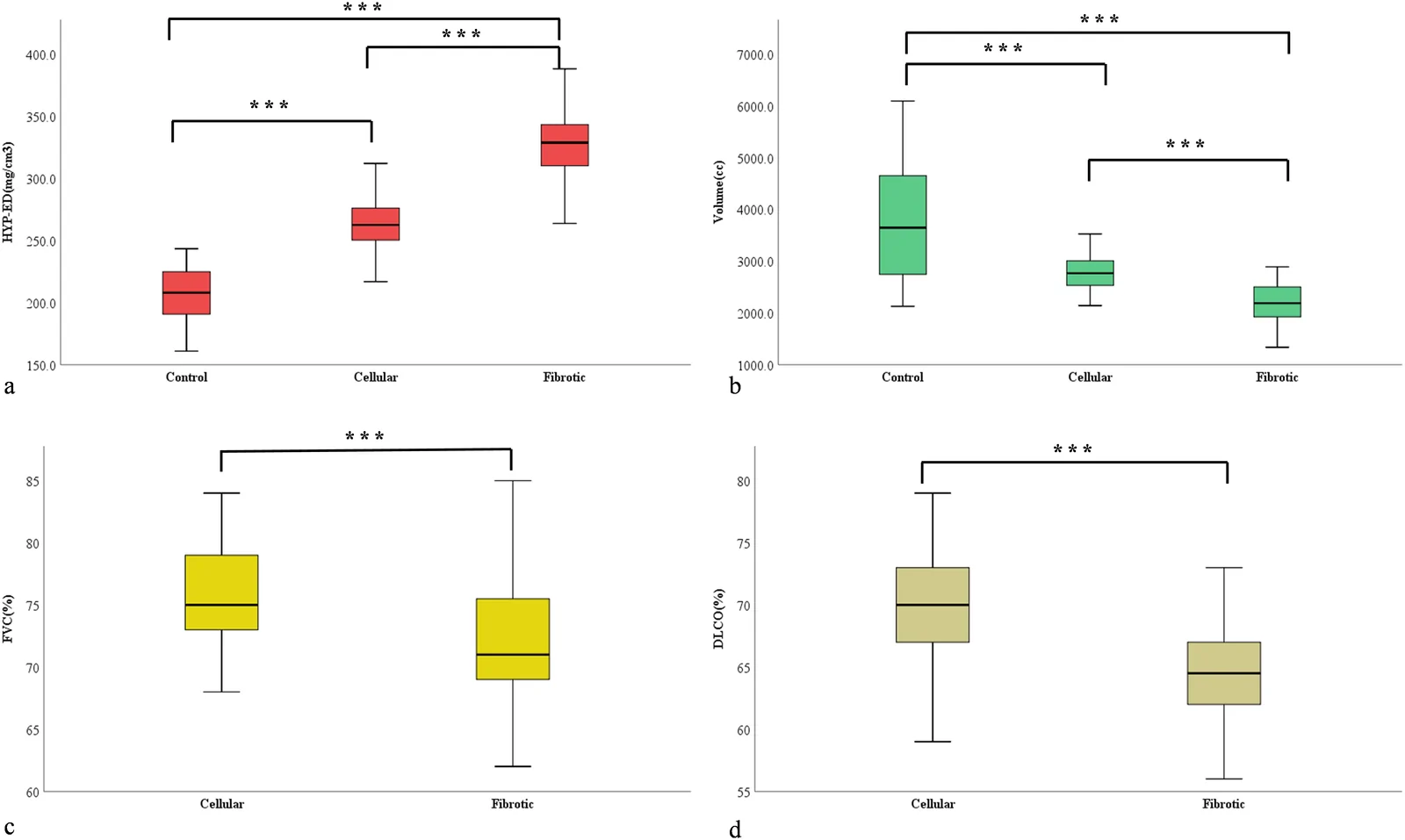
Comparison of key parameters across study groups. **(a)** HYP-ED. **(b)** Lung volume. **(c)** FVC% Pred in patients with cellular versus fibrotic NSIP. **(d)** DLCO% Pred in patients with cellular versus fibrotic NSIP. ***P < 0.001.

The HYP-ED in the control group measured 206.29 ± 22.40 mg/cm^3^, which was significantly lower than that observed in the cellular group (264.31 ± 24.99 mg/cm^3^, *P* < 0.001) and the fibrotic group (327.12 ± 36.26 mg/cm^3^, *P* < 0.001). Furthermore, a statistically significant difference (*P* < 0.001) was detected between the cellular and fibrotic groups (**Table 2**, **Figure 2a**).

**Table 2:** Comparison of HYP-ED content, lung volume, and pulmonary function parameters among different groups.

Parameter	Control (n=40)	Cellular (n=51)	Fibrotic (n=48)	F	*P*
HYP-ED (mg/cm³)	206.29±22.40	264.31±24.99	327.12±36.26	193.610	<0.001
Lung Volume (cc)	3797.40±1155.68	2796.88±334.03	2156.76±423.40	60.793	<0.001
FVC% Pred	/	75.98±4.07	71.44±5.49	22.051	<0.001
DLCO% Pred	/	69.96±4.41	64.58±3.38	45.885	<0.001

**Figure 3:**
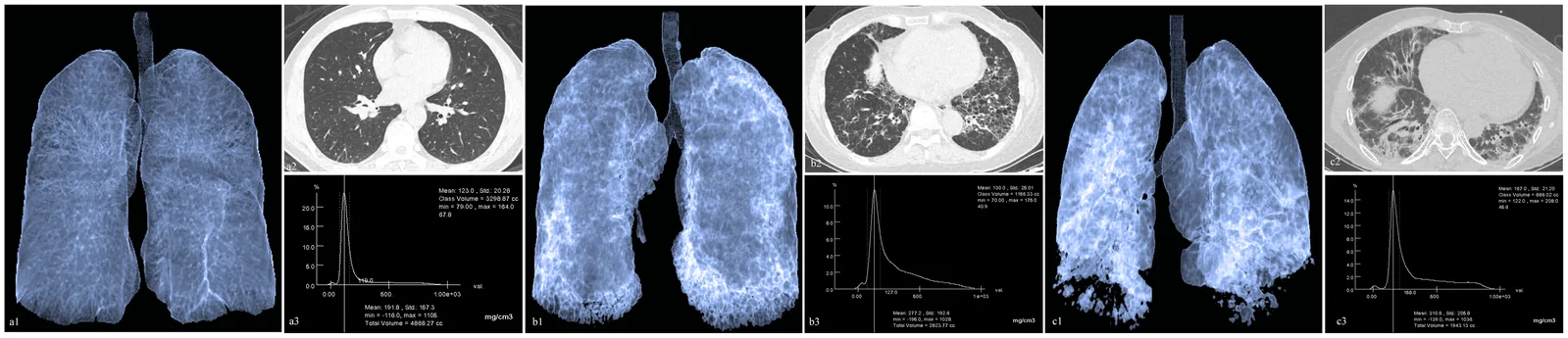
Representative cases from three groups. **(a)** Healthy control (female, 52 years): HYP-ED 191.8 mg/cm^3^, lung volume 4868.27 cm^3^. **(b)** Cellular NSIP (female, 54 years): HYP-ED 277.2 mg/cm^3^, lung volume 2823.77 cm^3^. **(c)** Fibrotic NSIP (female, 48 years): HYP-ED 310.6 mg/cm^3^, lung volume 1943.13 cm^3^. Panels: (1) volume-rendered (VR) image; (2) axial CT image; (3) HYP-ED distribution map.

Similarly, lung volume in the control group (3797.40 ± 1155.68 cc) was significantly greater than that in the cellular group (2796.88 ± 334.03 cc, *P* < 0.001) and the fibrotic group (2156.76 ± 423.40 cc, *P* < 0.001). Additionally, the cellular group exhibited a significantly higher lung volume compared to the fibrotic group (*P* < 0.001) (**Table 2**, **Figure 2b**). Three sets of case examples are shown in **Figure 3**.

### 3.3 Comparison of pulmonary function among different NSIP subtypes

The FVC% Pred in cellular NSIP patients was significantly higher (75.98 ± 4.07%) compared to fibrotic NSIP patients (71.44 ± 5.49%, *P* < 0.05) (**Figure 2c**). Similarly, the diffusing capacity of the DLCO% Pred was significantly greater in cellular NSIP patients (69.96 ± 4.41%) than in fibrotic patients (64.58 ± 3.38%, *P* < 0.05) (**Figure 2d**).

### 3.4 Correlation between HYP-ED, lung volume, and pulmonary function indices

**Figure 4:**
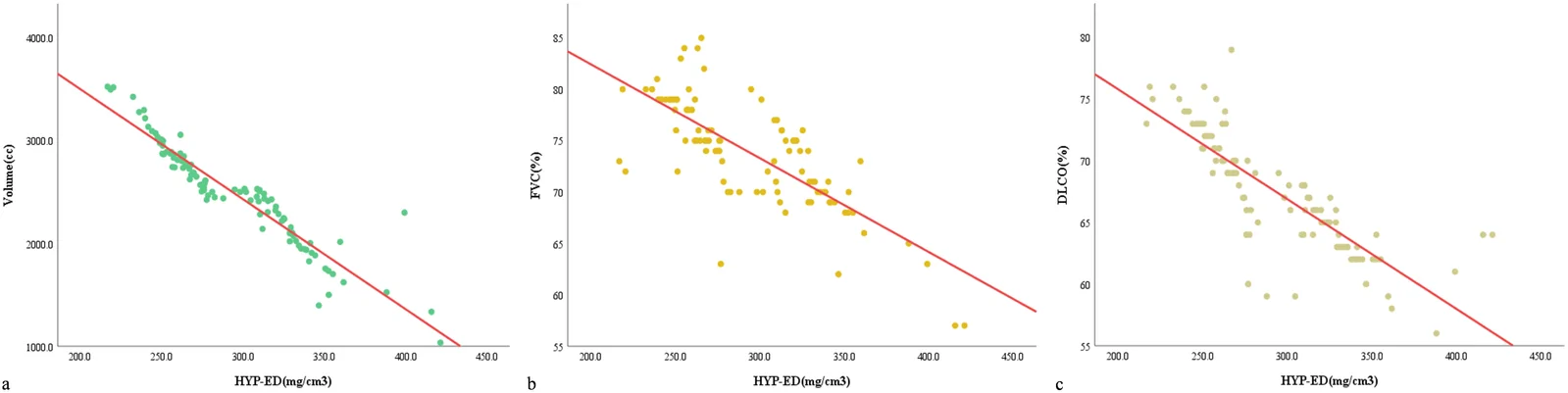
Scatter plots illustrating the correlations between HYP-ED and lung volume **(a)**, FVC% Pred **(b)**, and DLCO% Pred **(c)**.

Correlation analysis demonstrated a significant inverse association between HYP-ED and lung volume (Pearson r = −0.943, β = −11.63, *P* < 0.001), indicating that each 1 mg/cm^3^ increment in HYP-ED was associated with an average reduction of 11.63 mL in lung volume. A marginally significant interaction effect was observed between disease subtypes (*P* = 0.049), suggesting potential subtype-specific variations in this relationship (**Figure 4a**).

A significant negative correlation was identified between HYP-ED and FVC% Pred (Pearson r = −0.753, β = −0.124, *P* < 0.001), implying progressive deterioration in lung ventilation function with increasing HYP deposition. No significant interaction was detected between subtypes (*P* = 0.405), indicating a consistent association across both cellular and fibrotic subtypes of NSIP (**Figure 4b**).

Similarly, HYP-ED exhibited a significant inverse relationship with the percent predicted diffusing capacity of the DLCO% Pred (Pearson r = −0.813, β = −0.101, *P* < 0.001), supporting the notion that elevated HYP levels correlate with impaired gas exchange efficiency. A statistically significant interaction effect (*P* = 0.0001) suggested differential associations between HYP-ED and DLCO across subtypes (**Figure 4c**).

### 3.5 ROC curve analysis

**Figure 5:**
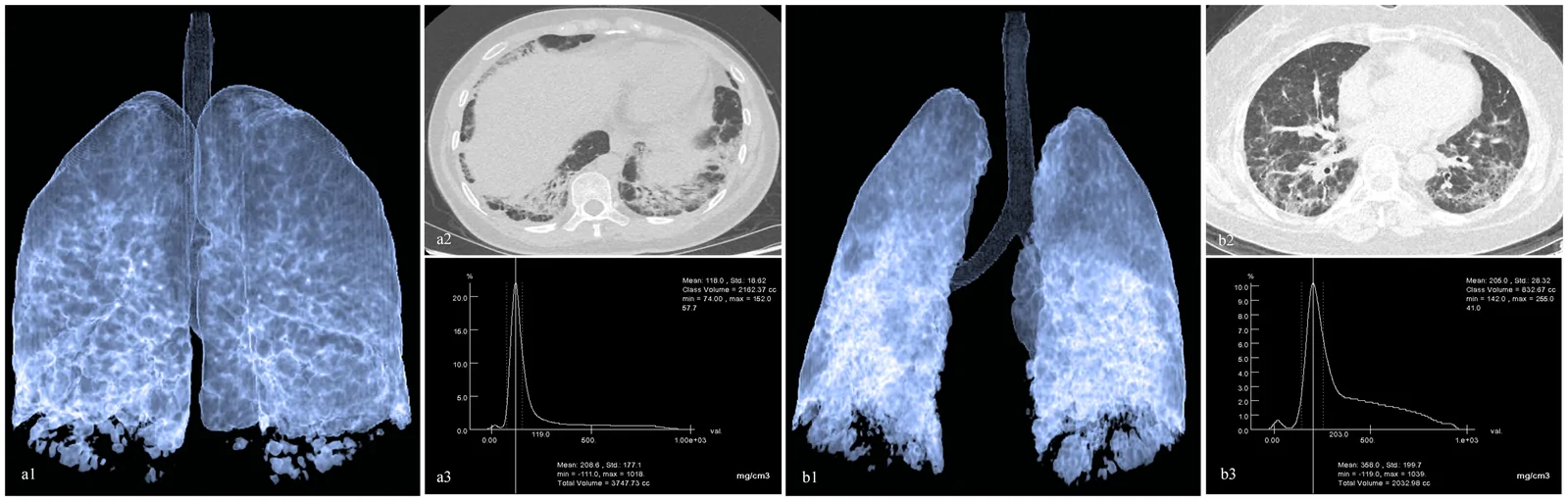
Representative imaging findings in NSIP. **(a)** Cellular NSIP (female, 49 years): HYP-ED 208.6 mg/cm^3^, lung volume 3747.73 cm^3^, FVC% Pred 82%, DLCO% Pred 80%. **(b)** Fibrotic NSIP (female, 53 years): HYP-ED 358.0 mg/cm^3^, lung volume 2032.98 cm^3^, FVC% Pred 70%, DLCO% Pred 65%. Panels: (1) VR image; (2) axial CT image; (3) HYP-ED distribution map.

**Figure 6:**
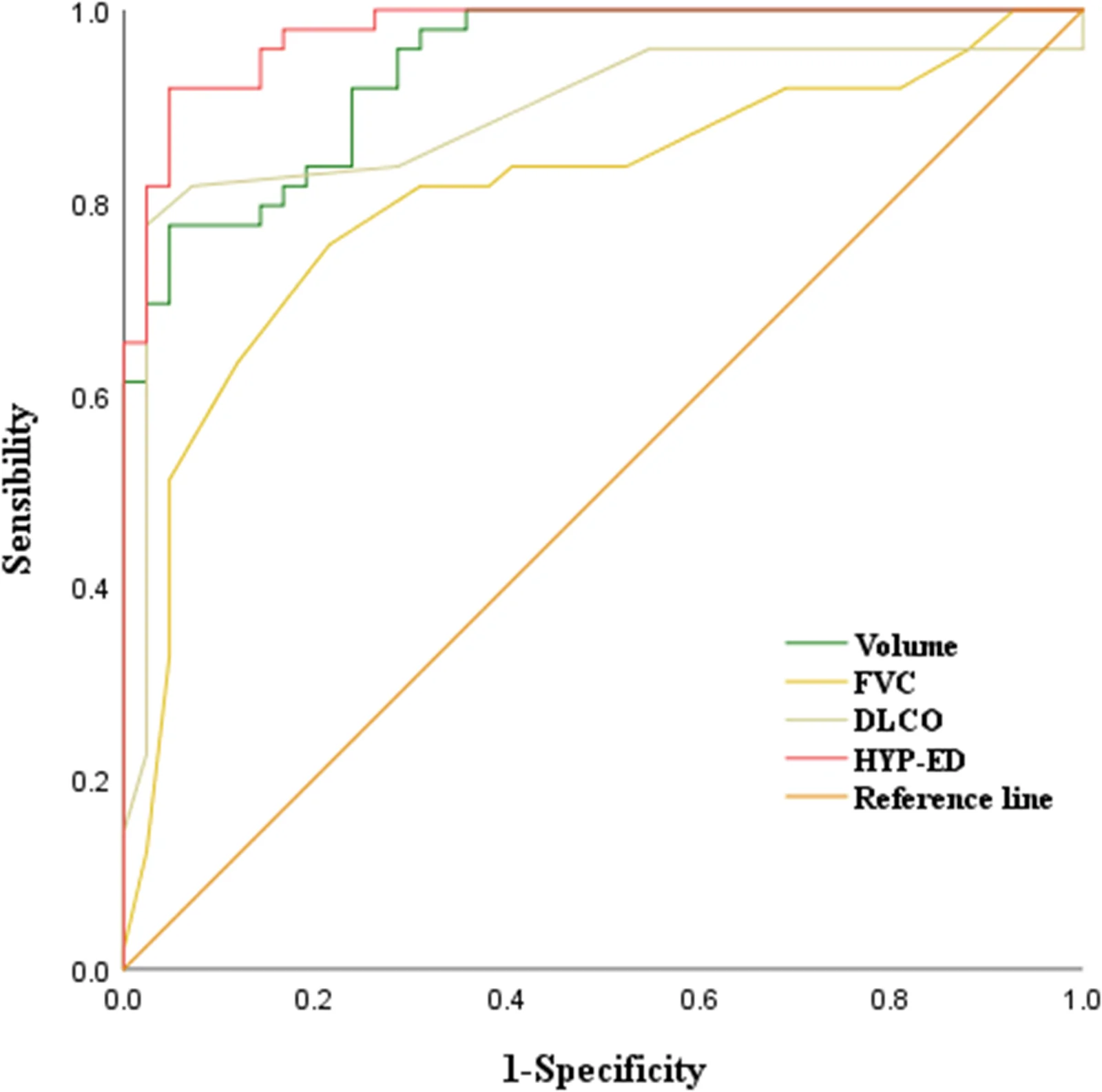
ROC curves for predicting fibrotic NSIP based on HYP-ED, lung volume, FVC% Pred and DLCO% Pred.

ROC curve analysis revealed that HYP-ED exhibited superior diagnostic performance in differentiating cellular from fibrotic NSIP, achieving an AUC of 0.977, which significantly surpassed the discriminatory capabilities of lung volume, FVC% Pred, and DLCO% Pred. At the optimal cutoff threshold of 296.6 mg/cm^3^, HYP-ED demonstrated high sensitivity (91.8%) and specificity (92.9%) (**Figure 5**). Although lung volume, FVC% Pred, and DLCO% Pred displayed moderate discriminatory potential, their overall diagnostic accuracy remained significantly lower than that of HYP-ED (**Table 3**, **Figure 6**).

**Table 3:** ROC analysis of HYP-ED, lung volume, FVC% Pred and DLCO% Pred for the diagnosis of fibrotic NSIP.

Parameter	Critical value	Sensitivity(%)	Specificity(%)	AUC
HYP-ED	296.6mg/cm³	91.8	92.9	0.977
Lung Volume	2537.6cc	77.6	95.2	0.939
FVC% Pred	75.5%	75.5	78.6	0.807
DLCO% Pred	66.5%	77.6	97.6	0.888

## 4 Discussion

Previous studies have demonstrated the utility of DECT with Fe_3_O_4_/water as a basis material pair for quantitative evaluation of Fe_3_O_4_ deposition in pulmonary tissues of arc welder’s pneumoconiosis patients, with *in vitro* validation confirming its reliability (12). Expanding upon this methodology, the current study introduces an innovative HYP/Ca material pair to facilitate quantitative measurement of HYP-ED in lung tissue. Our results revealed significantly higher HYP-ED in patients with NSIP compared to healthy controls, with particularly pronounced elevations observed in fibrotic NSIP cases. These findings indicate that HYP-ED quantification may serve as a potential biomarker for stratifying the severity of interstitial fibrosis. Notably, DECT-based HYP-ED measurement appears to correlate with collagen deposition levels in pulmonary tissue, thereby offering a novel, non-invasive imaging modality for the evaluation of pulmonary fibrosis progression.

In addition to HYP-ED, we observed a significant reduction in lung volume among NSIP patients relative to healthy controls, with the most marked decline occurring in fibrotic NSIP. Furthermore, fibrotic NSIP patients demonstrated significantly lower predicted FVC% Pred and DLCO% Pred values compared to cellular NSIP patients, suggesting progressive deterioration of lung volume, ventilatory capacity, and gas exchange efficiency with advancing fibrotic severity. These observations align with prior studies documenting diminished lung volume and declining pulmonary function in fibrotic lung disease (13). The observed lung volume reduction reflects diminished pulmonary compliance and structural remodeling, while decreased FVC% Pred and DLCO% Pred values signify restrictive ventilatory impairment and compromised alveolar-capillary gas transfer, respectively (14). Longitudinal investigations in bleomycin-exposed murine models have revealed persistent intracellular hydroxyproline accumulation despite ventilatory recovery, indicating sustained diffusion impairment (15). Additionally, clinical evidence suggests that FVC% Pred alterations may remain subclinical until lung volume decreases by 40–50%, implying that volumetric assessment may provide greater sensitivity than functional parameters for early disease detection (16).

Correlation analyses demonstrated significant inverse relationships between HYP-ED and pulmonary parameters including lung volume, FVC% Pred, and DLCO% Pred, which aligns with previous findings by Scharm et al. (17). These observations indicate that HYP-ED not only serves as an indicator of tissue-level structural remodeling but also correlates strongly with systemic functional decline. Consequently, DECT-based HYP-ED quantification could potentially integrate morphological and functional evaluations, thereby improving the clinical utility of imaging biomarkers.

In differentiating cellular and fibrotic NSIP, ROC analysis demonstrated that HYP-ED achieved an AUC of 0.977, outperforming lung volume, FVC% Pred, and DLCO% Pred. This finding has important clinical implications. Accurate identification of pathological subtypes is critical for guiding treatment strategies and prognostic evaluation. Moreover, in clinical scenarios where repeated lung biopsy is not feasible, a reliable non-invasive imaging biomarker capable of distinguishing disease subtypes could improve diagnostic efficiency while reducing patient burden.

Several limitations of this study should be acknowledged. First, this was a single-center retrospective study with a relatively small sample size, which may limit the generalizability of the findings. Second, the study focused exclusively on NSIP. Although this choice was justified by the continuous pathological spectrum from inflammation to fibrosis and the need to minimize confounding from diverse ILD subtypes, it inherently restricts the applicability of HYP-ED quantification to other fibrotic lung diseases; future studies should therefore include usual interstitial pneumonia (UIP) and other ILDs to verify generalizability. Third, two potential confounders warrant consideration. (1) Pharmacotherapy: 58 of 99 patients were receiving glucocorticoids, immunosuppressants, or antifibrotic agents at the time of CT, which may modulate inflammation and fibrosis and thereby influence measured HYP-ED levels. The proportion of treated patients did not differ significantly between cellular and fibrotic NSIP (54.9% vs. 62.5%, *P* = 0.443), minimizing the risk of asymmetric bias; however, the inclusion of both treated and treatment-naive patients introduces residual heterogeneity that cannot be fully adjusted. Future investigations should stratify by treatment regimen, enroll treatment-naive patients, or adjust for treatment duration to isolate the intrinsic DECT-derived HYP-ED. (2) Comorbidities and lifestyle factors: although conditions directly altering lung architecture were excluded, common comorbidities such as hypertension and diabetes mellitus, as well as smoking history, were not included as covariates. Their prevalence was well balanced across groups (all *P* > 0.05), limiting asymmetric confounding, yet subtle effects on HYP-ED quantification cannot be entirely excluded. Comprehensive comorbidity profiling and formal assessment of their independent impact are warranted. Fourth, although the HYP/Ca material pair was used for quantitative analysis, the accuracy and reproducibility of HYP-ED as a quantitative biomarker in lung tissue require further validation through *in vitro* experiments, pathological correlation, and multicenter studies. Fifth, pulmonary function parameters served as functional references, but systematic comparisons with histopathological fibrosis scores, clinical outcomes, and treatment responses were lacking. Therefore, future studies with larger prospective cohorts are needed to integrate pathological, serological, radiomics or texture analysis, and multimodal imaging data, while also accounting for treatment status and comorbidities, to establish a more robust model for evaluating fibrosis in NSIP and to extend DECT-derived HYP-ED quantification to other fibrotic ILDs.

In conclusion, this study demonstrates that DECT enables non-invasive quantification of HYP-ED in the lungs of NSIP patients. This parameter is significantly associated with lung volume and pulmonary function and shows high diagnostic performance in distinguishing cellular from fibrotic NSIP. DECT-derived HYP-ED quantification may serve as a promising imaging biomarker for assessing the severity of pulmonary fibrosis and monitoring disease progression in NSIP.

## Data Availability

The original contributions presented in the study are included in the article/supplementary material. Further inquiries can be directed to the corresponding authors.
